# Magnetoactive acoustic metamaterials based on nanoparticle-enhanced diaphragm

**DOI:** 10.1038/s41598-021-01569-9

**Published:** 2021-11-12

**Authors:** Xingwei Tang, Shanjun Liang, Yusheng Jiang, Cong Gao, Yujin Huang, Yuan Zhang, Chang Xue, Weijia Wen

**Affiliations:** 1 Department of Physics, The Hong Kong University of Science and Technology, Clear Water Bay, Kowloon, Hong Kong, China; 2 Division of Science, Engineering and Health Studies, College of Professional and Continuing Education, The Hong Kong Polytechnic University, Hung Hom, Kowloon, Hong Kong, SAR China; 3 College of Communication Engineering, Chongqing University, Chongqing, 400044 China; 4 Advanced Materials Thrust, The Hong Kong University of Science and Technology, Guangzhou, Guangdong China; 5 Shenzhen Fantwave Tech. Co., Ltd, Shenzhen, 518110 China; 6 Materials Genome Institute, Shanghai University, Shanghai, 200444 China; 7 HKUST Shenzhen-Hong Kong Collaborative Innovation Research Institute, Futian, Shenzhen, China

**Keywords:** Acoustics, Magnetic properties and materials

## Abstract

Magnetoactive membrane-type acoustic metamaterials are fabricated by coating a layer of magnetic nanoparticles on the polyethylene (PE) membranes and their vibration characters are investigated experimentally. From our experiments, we discovered that, under different magnetic fields by varying the distance between a magnet and the membranes, such membranes exhibit tunable vibration eigenfrequencies (the shift towards lower frequencies), which is caused by the variation of the effective mass density and effective tension coefficient resulted from the second derivative of the magnetic field. The strong magnetic force between the layer of magnetic nanoparticles and the magnet enhances the eigenfrequency shift. A spring oscillator model is proposed and it agrees well with the experimental results. We also experimentally observed that the vibration radius, effective mass density, and effective tension coefficient of the membranes can enormously affect the eigenfrequencies of the membranes. We believe that this type of metamaterials may open up some potential applications for acoustic devices with turntable vibration properties.

## Introduction

Membrane-type acoustic metamaterials (MAMs) have extremely thin and light properties that introduce a wide effective frequency regime and thus have a high application potential^1^. Initially, Yang et al.^2^ adjusted the vibrational eigenmodes by placing different small masses in the center of the membrane sample. Mei et al.^3^ then used an elastic membrane decorated with asymmetric rigid platelets to absorb low-frequency airborne sound at selective resonance. Decorated membrane resonators (DMRs) manipulate the pattern of membrane vibration or microstructures^4–10^ and apply an electric field^11–13^ or magnetic field^14–17^ to control the effective area, effective mass and effective tension coefficient of the membrane, demonstrating the infinite possibilities of MAMs^1,18–20^.

Magnetic field controlled metamaterials actively control the acoustic properties of metamaterials by introducing magnetic fields, which can control sound waves in more diverse ways^21–23^. The magnetic field alters the pattern of magnetic materials and has the ability to manipulate the microscopic mode^24,25^. Applying a magnet or electromagnet to use the first derivative of the magnetic field to vary the tension coefficient of the diaphragm, causing the eigenfrequency of the diaphragm to shift to higher frequencies^14,17,26^. The nonlinear coupling of magnetic field and force can also significantly improve the sound intensity produced by acoustic metamaterials^27^. As a result, incorporating magnetic fields into acoustic metamaterials optimizes maneuverability and promotes the development of acoustic metamaterials.

In this Letter, we demonstrate that by adjusting the relative position of a small magnet to the magnetic nanoparticle-enhanced diaphragm and the eigenfrequency of the diaphragm can shift towards lower frequencies, which results from the magnetic diaphragm experiencing varying magnetic field force while adjusting the distance between the magnet and diaphragm (the second derivative of the magnetic field), which manipulates the effective mass density and effective elastic coefficient of the diaphragm. The maximum frequency shift is close to $400~\text {Hz}$ when the initial eigenfrequency is around $1500~\text {Hz}$, due to the boost in magnetic force between the nanoparticle-enhanced membrane and magnet. An effective elastic vibration model is also proposed to describe the effective mass density evolution, which agrees well with the experimental results.

## Preparation and characterization of the membrane-type metamaterials

**Figure 1:**
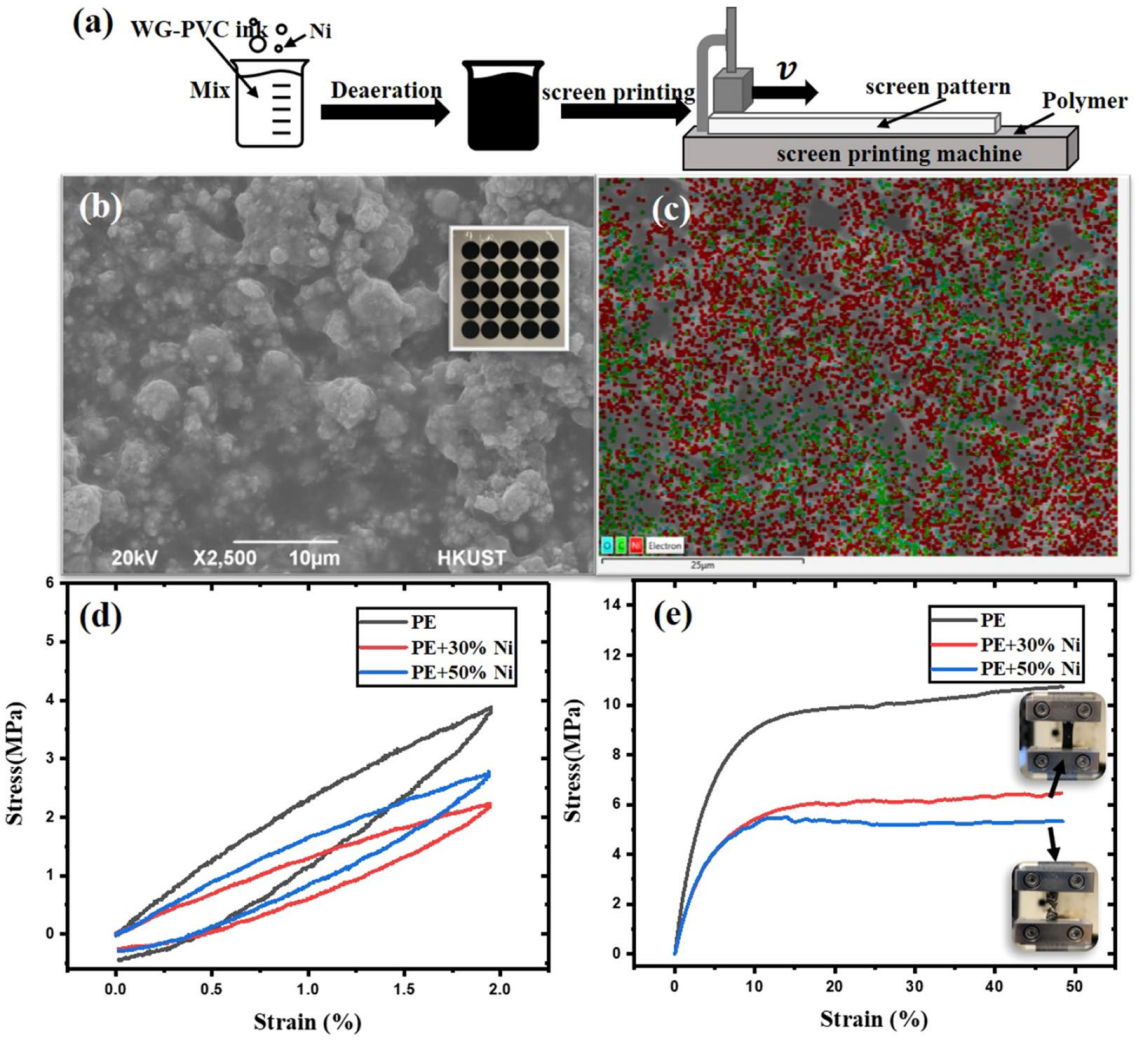
(**a**) Manufacturing process of the magnetic nanoparticle-enhanced diaphragm. (**b**) SEM image of the magnetic nanoparticle-enhanced diaphragm; the inset is the manufactured magnetic nanoparticle-enhanced membrane. (**c**) Elemental analysis of the surface of the magnetic nanoparticle-enhanced membrane; Ni is evenly distributed in the ink, and the red color represents Ni nanoparticles, while the green color represents the carbon element, which is the component of the ink. (**d**) Loop tensile test of different membranes: strain-stress curves. (**e**) Ultimate tensile test of different membranes: strain-stress curves. The insets show the deformation of 30% Ni printing PE and 50% Ni printing PE after 50% strain. It can be seen that the printing of 30% Ni is still well bonded to the PE membrane, but the 50% Ni printing has cracked on the PE membrane.

Figure 1a depicts the manufacturing method of the nanoparticle-enhanced diaphragm. The nano-nickel powder (Sigma-Aldrich) (<100 nm) and the water-based screen printing ink (Shenzhen Xinghuicheng Ink Technology Co., Ltd.) are evenly mixing in a specific mass ratio. After deaeration, the mixed paint is screen printed on a 20 μm thick PE membrane (Zetron Packaging). After allowing the coating to dry for a few hours, our nanoparticle-enhanced diaphragm is complete. The ink is wrapped with nanoparticles and attached to the PE membrane to enhance the magnetic properties of the PE membrane. Such as the enhancement of the magnetic force between the membrane and magnet, the improvement in relative permeability of the membrane. Actually, we could prepare magnetic membranes of varying thicknesses and shapes using different silkscreens, and the eigenfrequencies could be modified by adjusting the mass ratio, thickness and radius of the magnetic membrane. We prepared both a 30% and 50% mass ratio of magnetic nanoparticles and ink, respectively and the printing thickness is also 20 μm, resulting in a total thickness of PE plus coating of 40 μm. We prepared two types of membrane, each with a radius of 5 and 6 mm, respectively. A total of four circular magnetic coated PE membranes with different magnetic properties and radii were produced. The printed magnetic membrane is depicted in the inset of Fig. 1b. We observed the coating with nano-magnetic particles using scanning electron microscope (SEM), the nano-nickel powder is evenly wrapped by WG-PVC ink (Fig. 1b), and the Ni powder is evenly dispersed in the ink in Fig. 1c, where the red color represents Ni nanoparticles, and the green color represents the carbon element, which is the component of the ink. The distribution of nickel powder in the ink, on the other hand, is in a discontinuous phase, which introduces stress concentration points in the material, causing the material more brittle. As a result, 50% Ni is easier to crack than 30% Ni, and the stress decreases after cracking (Fig. 1e), while the inset shows that the 50% Ni coating cracked after 50% strain, whereas the 30% Ni coating remains well bonded to the PE membrane. Because there is no long range strength between the particles, the strength on nanoparticles is provided by the water-based ink, and the strength of the ink is not as strong as PE, so the membrane with magnetic nanoparticles will be softer (as shown in Fig. 1d,e). According to Fig. 1d, the Young’s modulus of the pure PE membrane is about $198~\text {MPa}$, while the magnetic membranes with 30% and 50% Ni have Young’s modulus of about $113~\text {MPa}$ and $142~\text {MPa}$, respectively.

**Figure 2:**
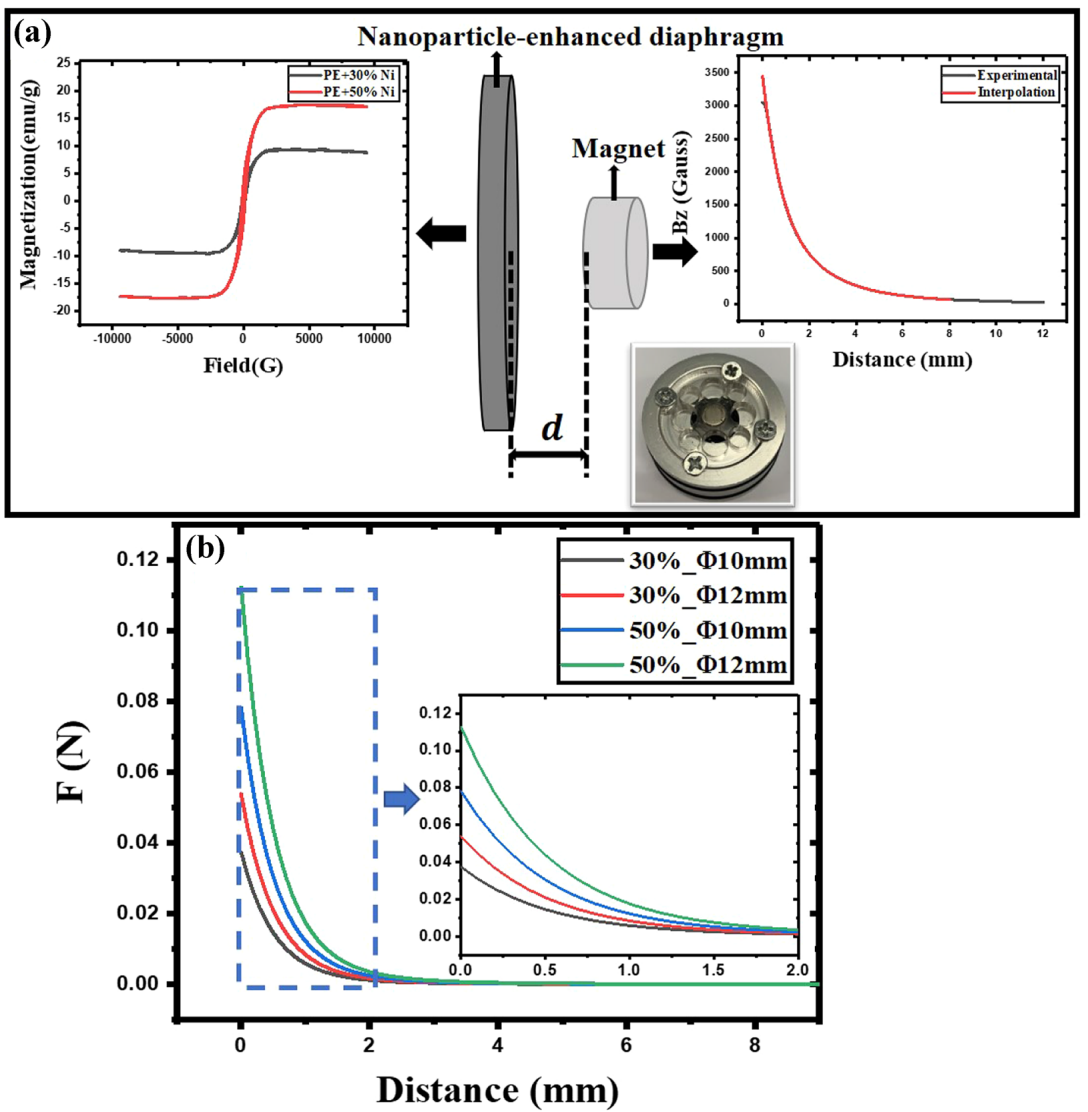
(**a**) The nanoparticle-enhanced diaphragm and the cylindrical magnet with a distance *d* from diaphragm, the lower right picture is the actual prototype used for testing. The left picture shows the vibrating-sample magnetometer (VSM) measurement results with different magnetic membranes, and the picture on the right shows the measured and interpolated values of the magnetic field at different positions of the magnet on its central axis. (**b**) When the cylindrical magnet has been determined to be a magnet with a magnetic field distribution on the right side of the (**a**), different nanoparticle-based diaphragms are subjected to magnetic forces at different distances from that magnet. The zoom in of the force applied to the magnetic membranes from 0 to 2 mm, respectively is shown in the inset.

The magnetization of magnetic membranes with different mass ratios is shown on the left side of Fig. 2a. The relative permeability $\mu _r$ of different magnetic membranes can be determined by $\vec{M}=\frac{\mu_r-1}{\mu_0\mu_r}\vec{B}$, where $\vec{M}$ is the magnetization, $\vec{B}$ is the magnetic field and $\mu _0$ is the vacuum permeability. The relative permeability of the 30% and 50% Ni magnetic membrane is approximately 1.07 and 1.17, respectively (refer to Appendix A of Supplementary Material), where the relative permeability of membrane is enhanced by larger nanoparticle mass ratio. The positional relationship between the magnet and the diaphragm is depicted in the middle of Fig. 2a. The central axis of the magnetic membrane coincides with the central axis of the magnet. The distance, *d* between them is adjusted (refer to Appendix B of Supplementary Material for the adjustment method). The magnet used here (Min Magnetic Company) is a strong neodymium iron boron, a cylinder with a radius of 2.25 mm and a height of 2 mm. The magnetic field of this magnet on the central axis is measured by a Gauss meter, as shown on the right side of Fig. 2a. However, the Gauss meter measurement is not particularly accurate, and the position of the Gauss meter probe relative to the central axis cannot be approximated as a point, in order to better calculate the magnetic field force, we have optimized the magnetic field of the central axis of the magnet by interpolating the measured value, and the force when the magnetic membrane is at different positions *d* from the magnet is obtained as follows (Fig. 2b):

1 $$\begin{aligned} \mathbf {F}&=-\nabla E=-\frac{\mu _r-1}{\mu _0\mu _r} V_{mem} \nabla B^2~, \end{aligned}$$

where *E* is the magnetic energy, and $V_{mem}$ is the membrane volume. It can be seen that the magnetic force received by the magnetic diaphragm is related to its relative permeability and size, but the overall trend is related to the magnetic field strength when the magnet is at different positions. The closer it is to the magnet, the greater the gradient of magnetic energy, and the greater magnetic force, but it can also be found that the magnetic force at a distance of about 0.9 mm from the magnet position dropped to 20% comparing with the force at distance between the magnet and the diaphragm is 0, while the magnetic field force at a distance of 1.3 mm is 10% comparing with the force at distance between the magnet and the diaphragm is 0, which gradually becomes even weaker.

**Figure 3:**
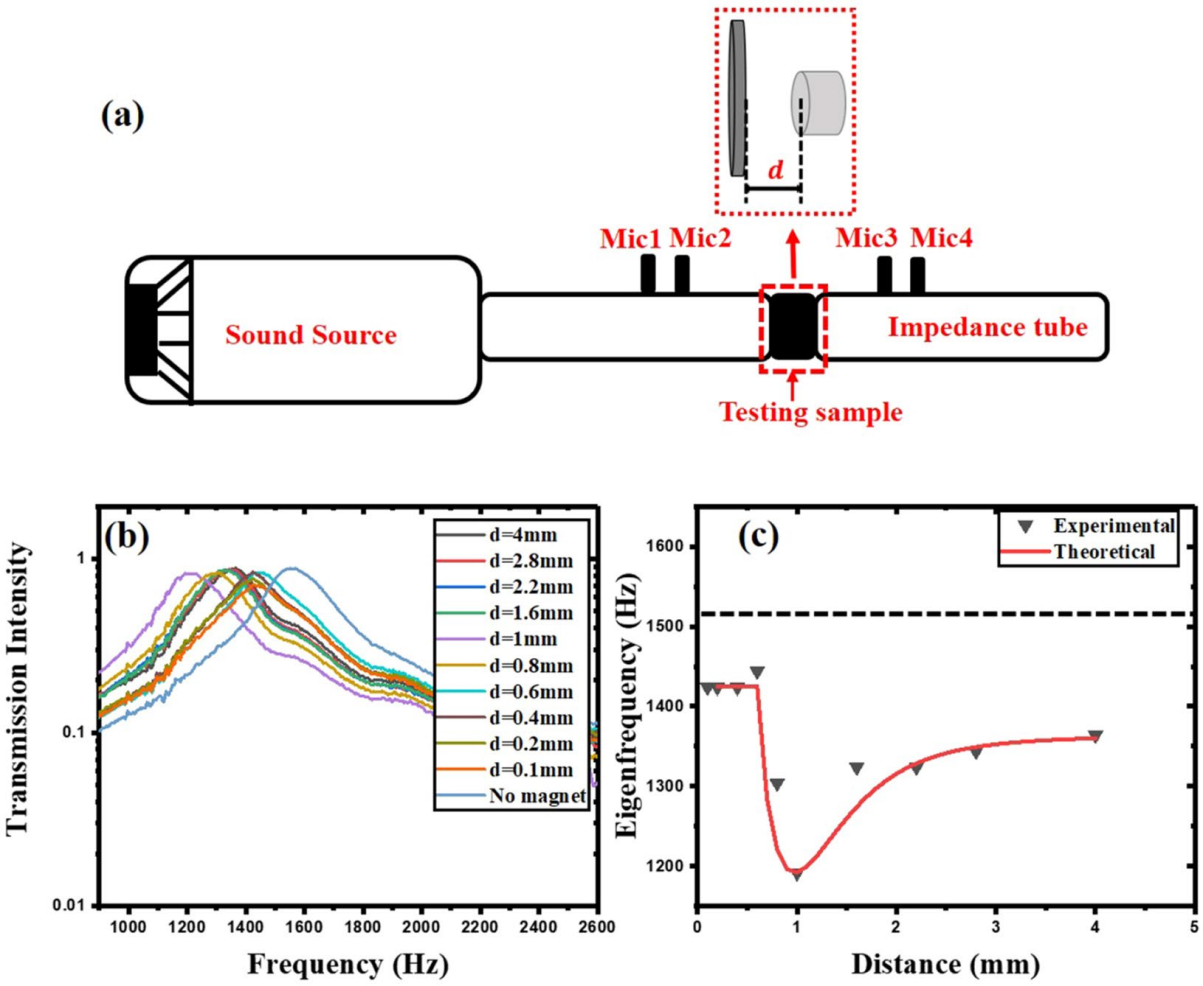
(**a**) Experimental set-up. The test sample (magnetic membrane and magnet installed on aluminum fixture) is placed into the impedance tube. (**b**) The transmission intensity spectrum of the diaphragm when the position of the magnet is adjusted differently away from the membrane. (**c**) The shift of the eigenfrequency of the membrane when the magnet is at different distances (the black dotted line is the initial fundamental eigenfrequency of the magnetic diaphragm).

The diaphragm is then fixed (the mass ratio is 50%, while the radius is 6 mm) in a non-magnetic aluminum fixture (fix the diaphragm in the fixture with a tension coefficient of 25 N/m, which can help maintain the position of the diaphragm even if the position of the magnet is changed). The magnet is installed on the aluminum fixture (Fig. 2a), and the assembled fixture is put in place (for dimensions of fixture refer to Appendix C of Supplementary Material) as a test sample into the impedance tube (Fig. 3a) to obtain the acoustical properties of the diaphragm at different magnet positions (Fig. 3b,c).

The transmission intensity spectrum of the membrane without magnet is first obtained, after which the eigenfrequency of the membrane can be obtained (the frequency at the maximum transmission intensity), which is in accordance with $f_n=\frac{\mu _n}{2 \pi r}\sqrt{\frac{T}{\sigma }}$, where $f_n$ is the $n$th eigenfrequency of circular diaphragm vibration, in this case, the fundamental eigenfrequency is used, $\mu _n$ is the $n$th zero point of the zero order Bessel function, *r* is the radius of the diaphragm, *T* is the tension coefficient of the diaphragm, and $\sigma$ is the area mass density of the diaphragm. The $\sigma$ of this diaphragm is $0.041\; \text{ kg}/\text{m}^2$. The magnet is then incorporated with an initial position of 4 mm away from the membrane. The frequency at the maximum transmission intensity of the membrane is observed to shift to lower frequency. The frequency shift increases as the magnet gets closer to the membrane. When it reaches 1 mm, the frequency shift towards the low-frequency reaches the maximum. Even if the magnet is moved closer to the diaphragm later, the variation of the fundamental eigenfrequency will not be greater than the frequency shift at 1 mm. We re-measured the transmission intensity spectrum of the diaphragm without magnet before and after adjusting the position of each magnet, and they all overlapped well. However, as the magnet gets closer to the membrane, the magnetic force on the membrane increases, and the membrane vibration is restrained, causing the transmission intensity at the fundamental eigenfrequencies of the diaphragm to gradually decrease (as shown in Fig. 3b). By taking the frequencies (the fundamental eigenfrequencies) of the maximum transmission intensity at different positions of the magnet, the shift trend of the fundamental eigenfrequencies of the diaphragm at different positions from the magnet is obtained (the triangular scatter points in Fig. 3c). We also tested the transmission intensity spectrum of the diaphragm for the diaphragms with different radii, different area mass densities (mass ratio). Then we can get their performances under different magnetic fields, and obtain their eigenfrequency under various conditions, all of which are in accordance with the expression $f_n =\frac{\mu _n}{2 \pi r}\sqrt{\frac{T}{\sigma }}$ (Appendix D of Supplementary Material).

## Results and discussion

**Figure 4:**
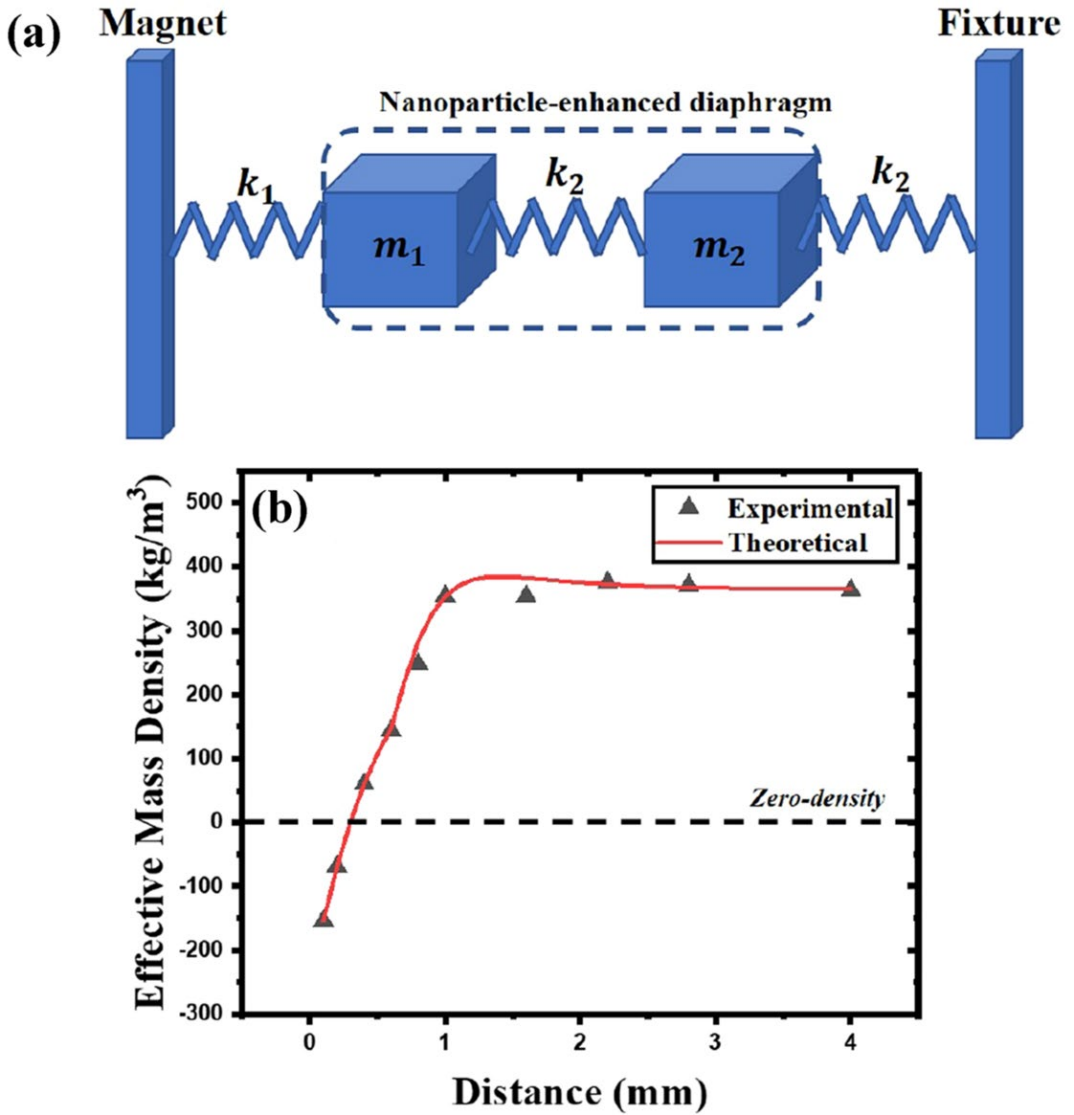
(**a**) Simplified model of magnet and magnetic nanoparticle-enhanced diaphragm. (**b**) Experimentally measured and theoretically calculated effective relative density of the diaphragm when the magnet is at different positions from the diaphragm (the black dotted line is the zero-density line).

To better analyze the magnetic nanoparticle-enhanced diaphragm and magnet system, we introduce spring vibrators (Fig. 4a). Because the cross-sectional area of the cylindrical magnet ($2.25^2\pi ~ \text{mm}^2$) is smaller than the surface area of the magnetic membrane ($6^2\pi ~ \text{mm}^2$), it does not cover the entire membrane. When the magnet is close to the diaphragm, the magnetic diaphragm is obviously divided into two parts, the circular area close to the magnet in the center of the membrane ($m_1$ in Fig. 4a) and the left circular ring area which does not receive too much magnetic force ($m_2$ in Fig. 4a). The force between $m_1$ and the magnet, which is dominated by a large magnetic force, is represented by a spring with an elastic coefficient of $k_1$, and because our diaphragm is fixed on the fixture, the fixture will also cause tension on the diaphragm. This tension is represented by a spring with an elastic coefficient of $k_2$, implying that the magnetic membrane is divided into two masses due to different magnetic forces. But they are always a whole membrane, and our default tension on the membrane is the same, so the elastic coefficient of the spring between $m_1$ and $m_2$ is the same as the elastic coefficient of the spring between $m_2$ and the fixture, which is $k_2$. Therefore, the entire nanoparticle-enhanced diaphragm becomes two masses ($m_1$ and $m_2$) connected in the middle by a spring with an elastic coefficient of $k_2$, and there are two springs with elastic coefficients $k_1$ and $k_2$, respectively connected with the magnet and the fixture(as shown in Fig. 4a). The introduction of the magnet causes the diaphragm to receive external force *F*(*x*), which can be expanded as $F(x) \approx F(x_0) + F^{\prime} (x)(x-x_0)$ around equilibrium position. Here, $F(x_0)$ changes the equilibrium position of the vibration and $F^{\prime}(x)$ changes the elastic coefficient of the diaphragm, thus the vibration equation of the entire nanoparticle-enhanced diaphragm is:

2 $$\begin{aligned} m_{eff} \frac{d^2x}{dt^2}+(k_2-F^{\prime}(x))x=0~, \end{aligned}$$

where $m_{eff}$ is the effective mass of the diaphragm, and $F^{\prime}(x)$ is the derivative of the magnetic force acted on the diaphragm when the distance between the magnet and the diaphragm is *x*, which is denoted as $k_1$ in Fig. 4a. Therefore we obtain:

3 $$\begin{aligned} m_{eff}=\frac{k_2-F^{\prime}(x)}{(2 \pi f)^2}~. \end{aligned}$$

Here, *f* is the eigenfrequency of the diaphragm. $k_2$ can be obtained from $F^{\prime}(x)=0$, the vibration equation when no magnet is added: $k_2=(\tilde{m}_1+\tilde{m}_2) \omega ^2$ ($\tilde{m}_1$, $\tilde{m}_2$ are the effective mass of two parts $m_1$, $m_2$ in the membrane), and we assume $k_{eff}=k_2-F^{\prime}(x)$, according to the equivalent lumped parameter of the circular membrane vibration^28^, at the fundamental frequency $\tilde{m}_1+\tilde{m}_2=(m_1+m_2)J_1 ^2(2.405)=0.27(m_1+m_2)=1.25~\text{mg}$, we denote $(\tilde{m}_1+\tilde{m}_2)$ as *M* in following calculation.

Therefore, we can obtain the effective mass $m_{eff}$ from the experimental data (the derivative of the magnetic force $F^{\prime}(x)$ can be obtained in Fig. 2b, $k_2=121.2~ \text{N}/\text{m}$, and the eigenfrequencies at each magnet distance in Fig. 3c). Then dividing by the volume of the membrane we get the effective mass density (the black triangle scatter points in Fig. 4b). There will be a negative effective mass density as the magnet gets closer to the diaphragm. This is due to the magnetic force received at a closer distance pulling the diaphragm’s center which causes center of membrane vibrate in the opposite direction. As the distance increases, the control (magnetic force) decreases, restoring the positive effective mass density.

Then we theoretically analyze this spring oscillator model. We assume that during the oscillation, the displacement of membrane’s mass center of the is *u*, the relative displacement of $m_1$ with respect to the mass center is $u_1$, the direction is left, while the relative displacement of $m_2$ with respect to the mass center is $u_2$, the direction is right, resulting:

4 $$\begin{aligned} \tilde{m}_1 u_1=\tilde{m}_2 u_2~. \end{aligned}$$

The equation of motion for $m_1$:

5 $$\begin{aligned} k_1(u-u_1)-k_2(u_1+u_2)=\omega ^2 \tilde{m}_1 (u-u_1)~, \end{aligned}$$

then for $$m_2$$:

6 $$\begin{aligned} k_2(u+u_2)+k_2(u_1+u_2)=\omega ^2 \tilde{m}_2 (u+u_2)~. \end{aligned}$$

For the nanoparticle-enhanced diaphragm as a whole:

7 $$\begin{aligned} (k_1+k_2)u+k_2 u_2-k_1 u_1=\omega ^2(u+u_2)m_{eff}~. \end{aligned}$$

Therefore, according to the definition of the effective mass^29^, the effective mass of the diaphragm can be expressed as:

8 $$\begin{aligned} m_{eff}=\frac{k_1+k_2}{\omega ^2}&\frac{u}{u+u_2}+\frac{k_2}{\omega ^2}\frac{u_2}{u+u_2}-\frac{k_1}{\omega ^2}\frac{u_1}{u+u_2}=\frac{k_2}{\omega ^2}+\frac{k_1}{\omega ^2}\frac{1-\frac{u_1}{u}}{1+\frac{u_2}{u}}~. \end{aligned}$$

Combining Eqs. (4), (5), (6), we get $u_1/u=\frac{(\tilde{m}_1 \omega ^2+\tilde{m}_2 \omega ^2-k_1-k_2)}{k_2 \tilde{m}_1 -k_1 \tilde{m}_2}$, $u_2/u=\frac{\tilde{m}_1(\tilde{m}_1 \omega ^2+m_2\omega ^2-k_1-k_2)}{k_2 \tilde{m}_1-k_1 \tilde{m}_2}$, and then applying them into Eq. (8), we get:

9 $$\begin{aligned} m_{eff}=\frac{k_2}{\omega ^2}+\frac{k_1}{\omega ^2}\frac{k_2 M-\tilde{m}_2 M\omega ^2}{\tilde{m}_1 M\omega ^2-k_1M}~. \end{aligned}$$

According to the experimental results, we know that $\omega <\omega _0=\sqrt{\frac{k_{2}}{M}}$, we set $\omega =\alpha \omega _0=\alpha \sqrt{\frac{k_2}{M}}$, and $\alpha <1$. In addition, we denote $\tilde{m}_1/M$, $\tilde{m}_2/M$ as $\eta _1$, $\eta _2$, respectively, where $\eta _1<1$, $\eta _2<1$, and $\eta _1+\eta _2=1$. Therefore, Eq. (9) becomes:

10 $$\begin{aligned} m_{eff}=M\frac{k_1\eta _2-k_2\eta _1}{k_1-\alpha ^2k_2\eta _1}~. \end{aligned}$$

By putting $M=0.27(m_1+m_2)=1.25~ \text{mg}$, $k_1=F^{\prime}(x)$, $k_2=121.2~ \text{N}/\text{m}$, $\alpha =0.87$, and the function $\eta _2$ (refer to Appendix E of Supplementary Material for the function plot), the theoretical effective mass can be obtained, and then by dividing the membrane volume, the effective mass densities can be obtained (the red solid line in Fig. 4b), which are in line with the experimental values. Here, we should pay attention to the valid domain of Eq. (10), where the $k_1$ is large enough to divide the membrane into two parts, which implies the occurrence of the effective mass transfer between $\tilde{m}_1$ and $\tilde{m}_2$.

Combining Eqs. (3) and (10), we can theorize that the eigenfrequency of the diaphragm when the magnet is at different positions is:

11 $$\begin{aligned} f(d) = \frac{1}{2 \pi } \sqrt{\frac{(k_2 - k_1(d))(k_1(d) - \alpha ^2 k_2 \eta _1(d))}{M(k_1(d)\eta _2(d) - k_2 \eta _1(d))}}~. \end{aligned}$$

The theoretically obtained eigenfrequency function in Fig. 3c plotted are in line with the experimental values.

## Conclusion

In summary, the eigenfrequency of the magnetic diaphragm can be controlled by adjusting the distance between the magnet and the magnetic diaphragm. With different positions of the magnet, the derivative of the magnetic force can be used to manipulate the effective elastic coefficients and effective mass density of the membrane, resulting in a shift of eigenfrequency. The frequency shift is also enhanced by the layer of magnetic nanoparticle in the membrane, due to the strong magnetic interaction between the magnet and the magnetic diaphragm. The magnetoactive acoustic metamaterial, which is made of a magnetic membrane, is not only simple to manufacture and control, but it can also realize eigenfrequency diversity, resulting in better sound wave control.

## Supplementary Information

Supplementary Figures.

## Data Availability

The data that support the findings of this study are available from the corresponding author upon reasonable request.
