# Supplementary material for: Magnetoactive acoustic metamaterials based on nanoparticle-enhanced diaphragm
Source: Sci Rep. 2021 Nov 12;11:22162. doi: 10.1038/s41598-021-01569-9 (PMC8589973; doi:10.1038/s41598-021-01569-9)
Supplement: Supplementary file 1 — Supplementary Figures. [file 41598_2021_1569_MOESM1_ESM.docx]

**Magnetoactive acoustic metamaterials based on nanoparticle-enhanced diaphragm**

**Supplementary Material**

**Appendix A.**

**
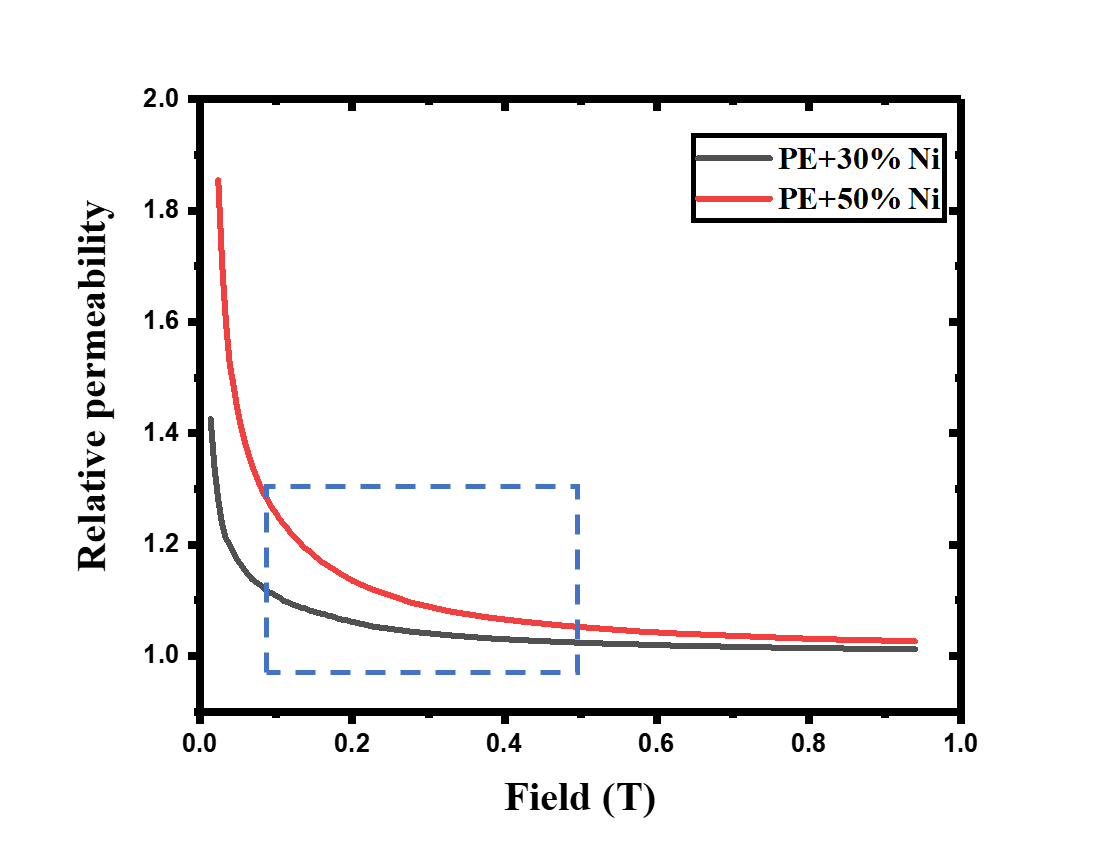
**

.**FIG. S1** The relative permeability of different magnetic membranes under different magnetic fields

For the relative permeability of different magnetic membranes, we use the smooth region of magnetic material to get an effective permeability.

**Appendix B.**

**
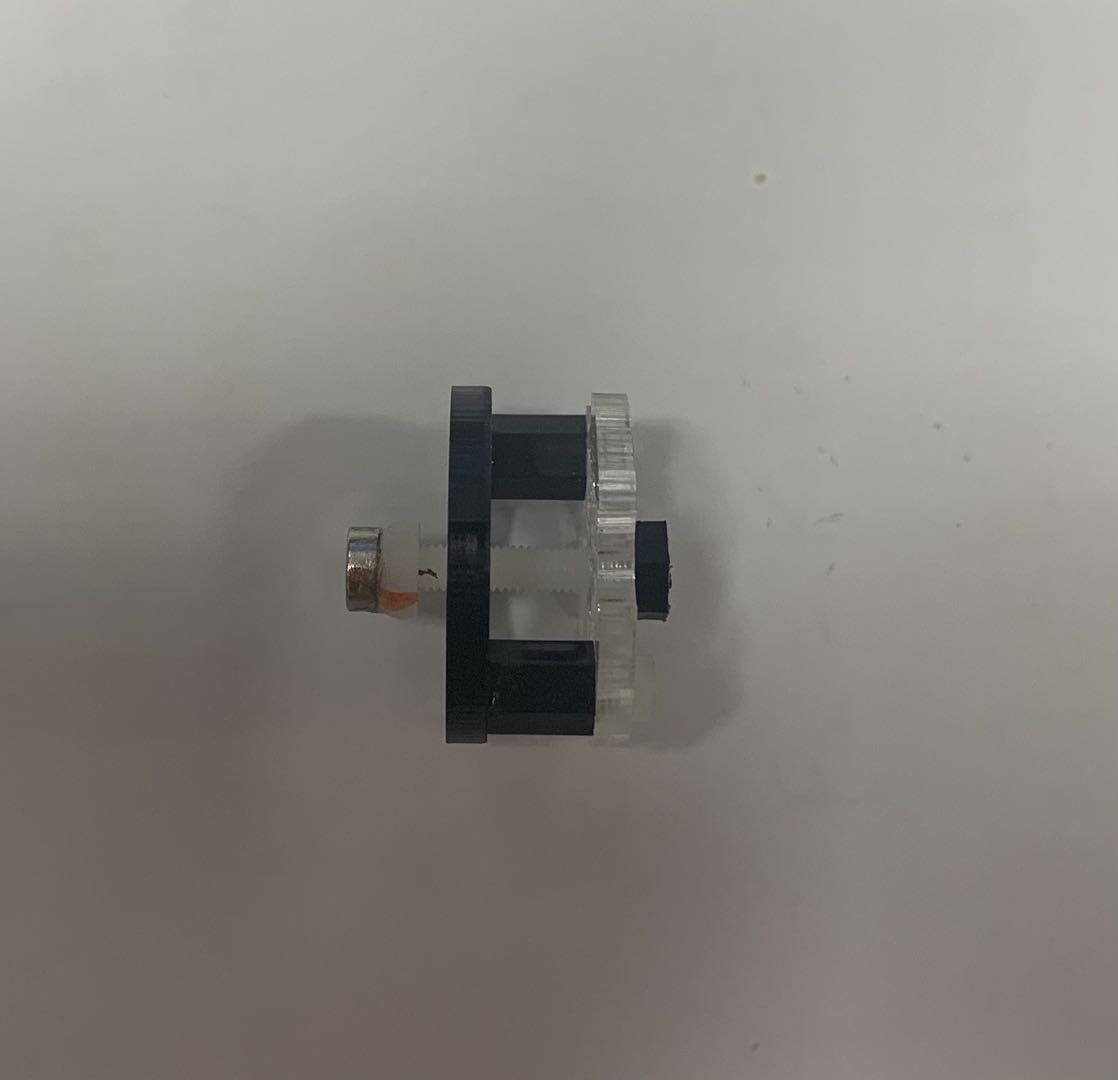
**

**FIG. S2** Device used to adjust the distance of the magnet

Fix the magnet on a non-magnetic screw, and the screw moves 0.6mm forward or backward by turning the screw one turn. Therefore, the moving distance of the magnet can be controlled with higher accuracy by rotating the screw at different angles.

**Appendix C.**


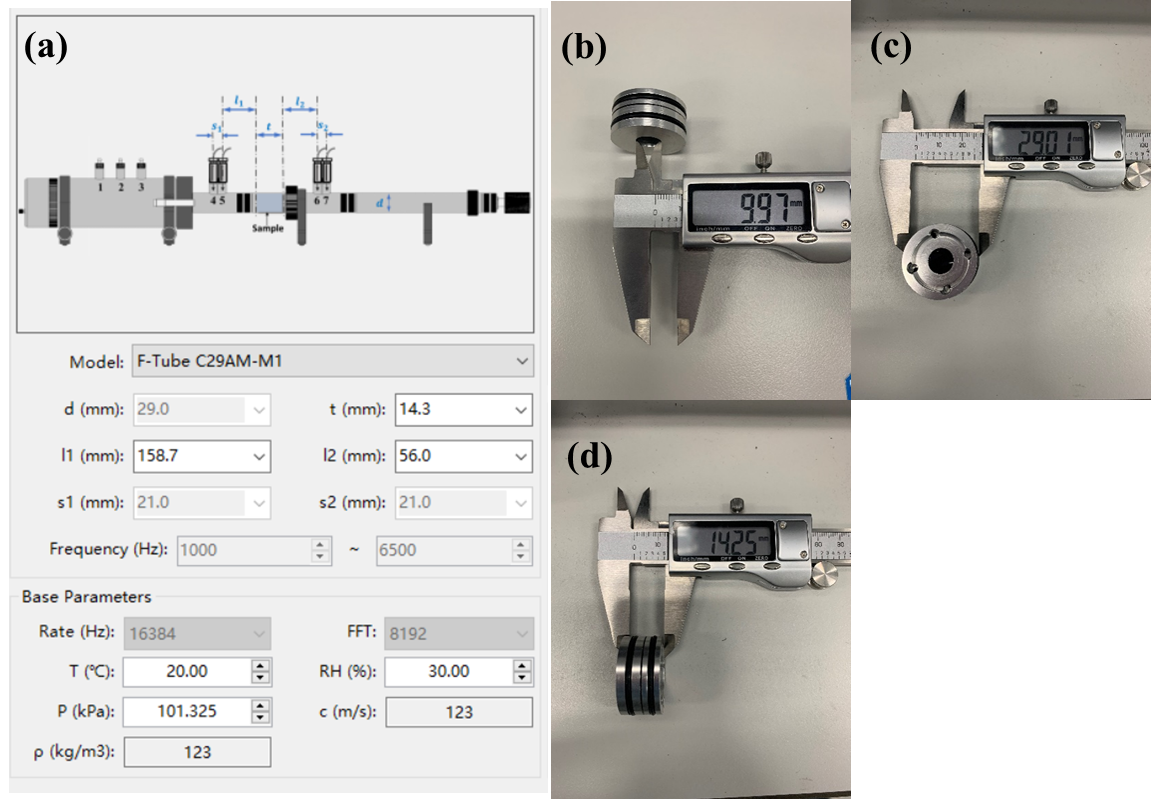


**FIG. S3** (a) The size of impedance tube and the distribution of microphones. (b) (c) (d) Dimensions of testing samples

**Appendix D.**


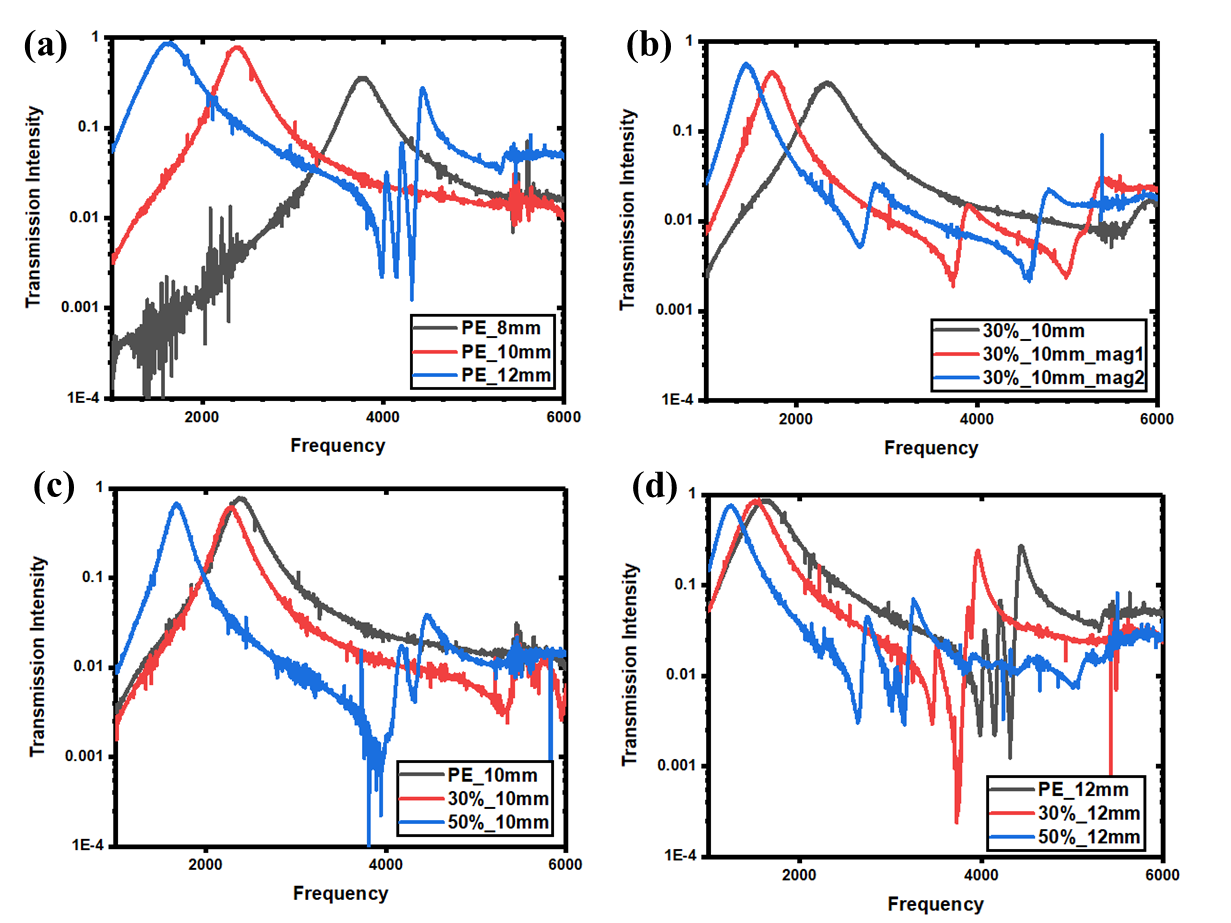


**FIG. S4** (a) The transmission intensity spectrum of PE membranes with different radii. (b) The transmission intensity spectrum of 30% Ni printing magnetic membranes with a diameter of 10 mm under different external magnetic fields. (c) The transmission intensity spectrum of the membranes with a diameter of 10mm with different surface mass densities. (d) The transmission intensity spectrum of the membrane with a diameter of 12mm with different surface mass densities.

**Appendix E.**

**
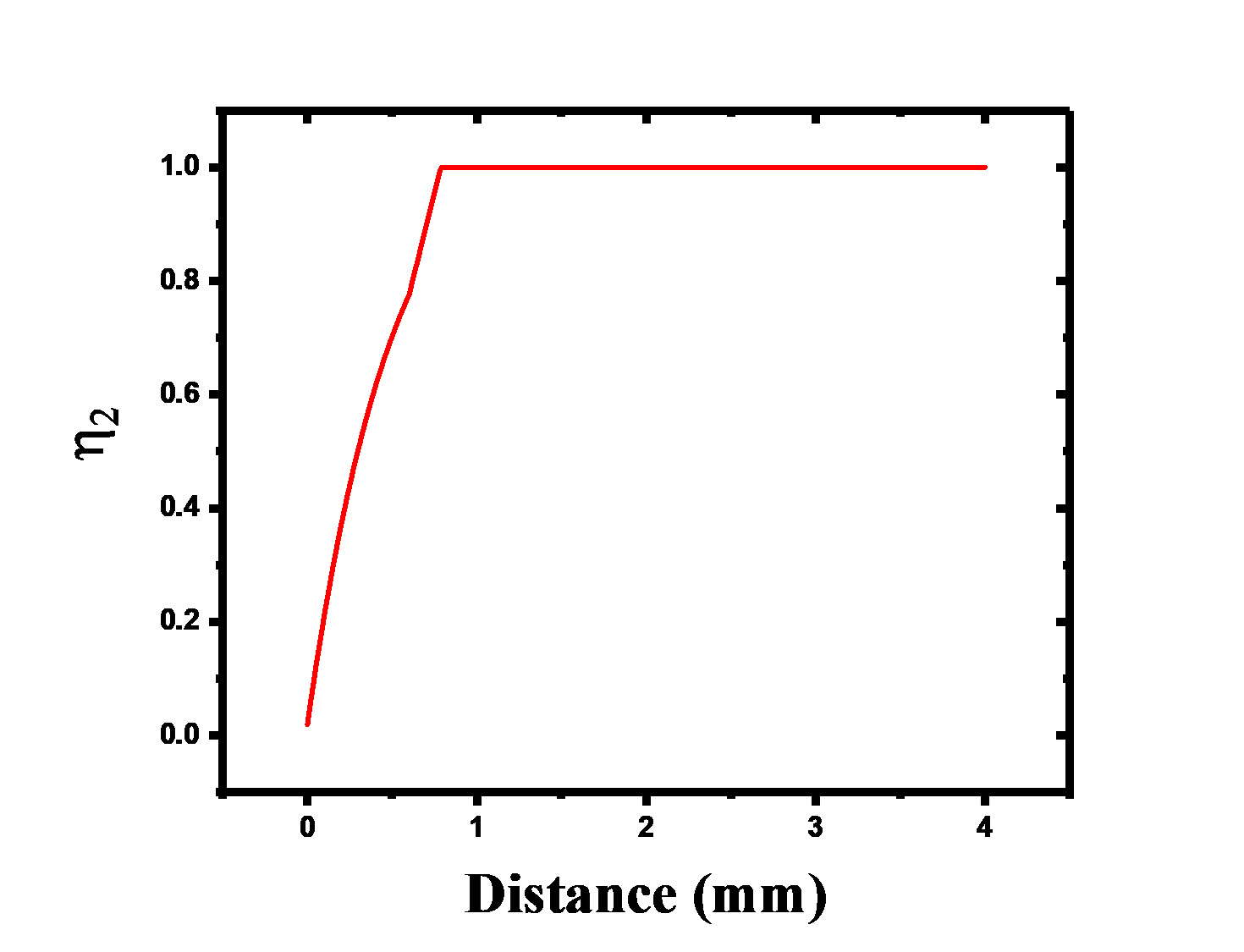
**

**FIG. S5** The relationship between $\eta_{2}$ and distance
